# Towards the Reuse of Fire Retarded Polyamide 12 for Laser Sintering

**DOI:** 10.3390/ma17164064

**Published:** 2024-08-15

**Authors:** Dylan Seigler, Marcos Batistella, José-Marie Lopez-Cuesta

**Affiliations:** Polymers Composites and Hybrids (PCH), IMT Mines Ales, 30319 Ales, France; dylan.seigler@mines-ales.fr (D.S.); jose-marie.lopez-cuesta@mines-ales.fr (J.-M.L.-C.)

**Keywords:** polyamide 12, SLS, fire-retardant, material characterization, aging

## Abstract

The control of powder aging during Selective Laser Sintering (SLS) processing is one of the challenges to be overcome for the implementation of this technique in serial production. Aging phenomena, because of the elevated temperatures and long processing times, need to be considered when a fraction of the polymer powders present in the build chamber and not used to manufacture the parts are reused at various times. The aim of this study was to investigate the influence of successive reuse of blends of pure Polyamide 12 and its blends with two types of flame retardants (FR): ammonium polyphosphate (APP) and zinc borate (ZB). The composition of the blends was 70/30 (wt/wt) PA 12/FR. Four successive processing stages have been carried out by collecting the remaining powder blend each time. The powders were re-used using the same processing parameters after sieving. DSC measurements showed that the incorporation of FRs entailed a reduction in the processing window up to 4 °C; nevertheless, no further reduction was noted after aging. The TGA curves of aged blends of powders were also similar for pure PA 12 and PA 12 with FR. In addition, initial and reused powders presented a higher degree of crystallinity than the specimens processed from the powders. The heterogeneous character of the PA 12 after LS processing or reprocessing was shown through Pyrolysis Combustion Flow Calorimetry (PCFC) and cone calorimeter (CC) tests. FTIR analysis also showed that post-condensation reactions have occurred. The mode of action of the flame retardants was clearly seen on HRR curves at both tests. The first reuses of PA 12 powders entailed a significant reduction in time to ignition at the cone calorimeter (150 for the initial material to around 90 s for the reused material), indicating the formation of short polymer chains. Only in the case of zinc borate was it noticed that re-used powder was detrimental to the fire performance because of a strong increase in the value of pHRR (between 163 and 220 kW/m^2^ for reused material instead of 125 kW/m^2^ for the initial one).

## 1. Introduction

Additive manufacturing is growing in terms of technologies and applications. Among the various AM technologies, Laser Sintering (LS) attracted attention in the literature because of its ability to build parts with very complex geometries [1,2]. Nowadays, various polymer powders are available for LS, such as polyamides (6, 11, and 12), polyether ether ketone, polyether block amide copolymers, and polyurethanes [3,4]. Among these, polyamides are the most used because of their unique properties and ease of processing [5]. However, one of the drawbacks of polyamides is the aging of the powder during the processing stages, which limits its recyclability [6]. Powder recycling in LS by collecting and reusing unused powder from previous prints is necessary, owing to the cost of polymer powders as well as the need to save material for environmental concerns. Hence, after printing, the powder is cleaned, sieved, and mixed with fresh powder to create a new feedstock for the next LS process.

Various works in the literature have evaluated the influence of recycling PA 12 in terms of powder characteristics (molar mass, crystallinity, melting, and crystallization temperatures) as well as its influence on printed parts (mechanical properties, porosity) [6]. Furthermore, the influence of oxygen content on the build chamber was also evaluated, and thermo-oxidation effects were highlighted [7,8,9]. Assessing the influence of thermo-oxidation is important because oxygen always remains in the build chamber.

During the LS process, the powder is subjected to thermal stress, which is determined by the processing time and temperature and can lead to a physicochemical degradation of the polymer (agglomeration, chain-scission, and crosslinking) [6,9,10,11,12]. Various investigations on aged PA 12 powder showed an increase in average molar mass due to thermal-induced post-condensation reactions. Pham et al. [13] evaluated the influence of storage time and temperature on the melting and crystallization temperatures and the average molar weight. A decrease in the crystallinity and an increase in the melting temperature were observed with an increase in the storage time, which was attributed to post-condensation reactions. Polyamide chains are linked by amide groups, though the units normally contain some open chain ends, which can go through a post-condensation and crosslinking reaction, especially at higher temperatures [8].

The influence of aging on the quality of sintered parts (most mechanical and surface rugosity) was also extensively evaluated in the literature [9,14,15,16,17]. Various works assessed the effect of reuse and build time on shape and size distribution as well as the morphology of PA 12 feedstock powders [16,18]. The term “build time” refers to the periods of time for which the polymer powder undergoes a temperature higher than room temperature during the following stages of the LS process. This “build time” includes the pre-heating time, the time for the machine to rise to the printing temperature, the time to melt each layer of the part, the time left between layers during laser irradiation, and the time for the part to be cooled completely before being removed from the building chamber.

It has been shown by Alo et al. [19] that particle size distributions of the powders increase with the proportion of fine particles at higher reuse cycles. This phenomenon was attributed to cracking and fragmenting during repeated exposure to the high processing temperature during LS. Furthermore, an increase in the orange peel morphology and a deviation of part dimensions were observed for higher building times. Dotchev [6] evaluated the influence of the refreshing strategy, build height, powder position, and aging state of PA 12 through variations in the melt flow rate (MFR).

Beyond the degradation observed on powder particles, various works evaluated the influence of reuse and related building time on mechanical properties. However, the results reported in the literature were not consistent. Earlier works of Zarringhalam showed that reuse had minimal influence on ultimate tensile strength (UTS) [20], whereas more recent works found a significant decrease in UTS and an increase in ductility [18]. These changes were attributed to an increase in the molar weight, leading to an increase in viscosity and the formation of residual voids that act as local stress concentrators [21]. Furthermore, the use of different grades of PA 12 and printers could also explain different panels of properties. Yao et al. [22] compared the tensile strength of PA 12 recycled eight times and found an increase in the tensile strength for the first reuse compared with virgin PA 12 and a gradual decrease from the first to the eight-reuse cycle. This behavior of eight times reused was explained by an increase in PA 12 crystallite size in comparison with neat PA 12 [22].

Beyond mechanical properties, various applications, such as transports, need parts with increased flame retardancy. The main strategy to improve the fire reaction of polymers processed through LS is the blending of polymer powders with flame retardants available as powders of relevant particle size distribution. Recent works showed that flame retardants must be carefully chosen in order to obtain fully dense parts for flame retarded PA 12 [23,24]. In particular, the use of flame retardants could affect the thermo-optical properties of the powder, affecting its reuse. Moreover, specific attention has to be drawn to the rheological behavior of the powder blend.

To the best of our knowledge, no work has been carried out evaluating the influence of the reuse of blends of PA 12 powders with flame retardants on the conservation of fire performance. Thus, the objective of this work is to assess the thermal and chemical properties of the powder blends of PA 12 filled with two types of flame retardants (ammonium polyphosphate (APP) and zinc borate (ZB)) after successive LS processing cycles. The role of APP in the improvement of the fire reaction of aliphatic polyamides is well known [25]. Zinc borate is rather used as a synergistic agent for polyamides [26], but its interest was also emphasized in our previous work about flame retarded PA 12 processed through LS [23]. Particular attention is focused on the characterization of the powders as well as the influence of the aging of powders on the processing ability. Moreover, the investigations also concern the fire performance of materials processed with the use of recycled blends at different aging stages. In particular, the fire behavior of filled PA 12 with flame retardants after different reuse cycles is studied through cone calorimeter testing.

## 2. Materials and Methods

**Figure 1:**
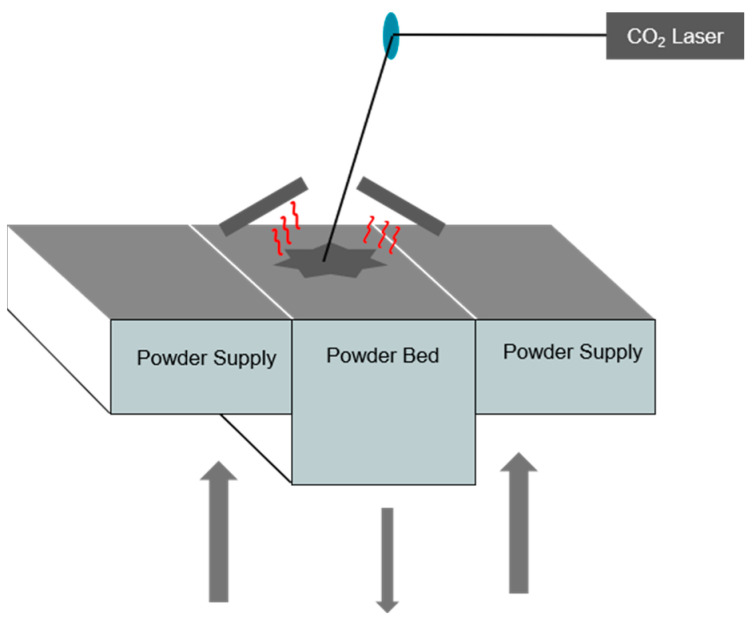
SLS printer operating principle.

Selective laser sintering (Figure 1) uses a high-power laser (CO_2_) to melt polymer powder particles to create a solid part based on a 3D model. The wavelength used in the SLS process is linked to the emission wavelength of the CO_2_ laser and is 10.6 µm.

The first stage of the process involves depositing a thin layer of polymer powder in the printing tray (building area). A roller or blade is then used to spread the powder and supply powder to the build area from the tanks located on either side of the manufacturing chamber. These powder tanks contain additional powder in stock (powder supply). The powder bed is then pre-heated to a temperature above the crystallization temperature and close to the melting temperature of the polymer, defining the sintering window. Then, the laser selectively melts the areas to be sintered, layer by layer, with the aim of producing the part from the bottom to the last layer at the top.

PA 12 (PA2200) powder with a median diameter (D50) of 58 µm was supplied by Electro Optical System (Krailing, Germany). Based on a previous work [23], two flame retardants were selected: ammonium polyphosphate (Exolit AP 422 from Clariant, D_50_ = 17 µm) and zinc borate (2ZnO•3B_2_O_3_•3.5H_2_O, Firebrake ZB from Rio Tinto Minerals D_50_ = 9 µm). Both FRs were added at 30%wt. using a powder mixing station P1 from EOS, Germany. The following abbreviations for each additive were used: AP30-PA 12 + 30 wt% of ammonium polyphosphate and ZB30 PA 12 + 30 wt% of zinc borate, followed by the cycle number: 0 for virgin powder, 1 for the powder after the first reuse and so on.

Test specimens (sheets of 70 × 70 × 4 mm^3^) were produced with an LS printer (SnowWhite from Sharebot, Nibionno, Italy). The same parameters were used for all compositions. The parameters are shown below:

- Warm-up temperature: 151 °C
- Printing temperature: 153 °C
- Laser scan speed: 2.85 m·s^−1^
- Laser power: 3.64 W
- Layer height: 0.1 mm

The bed temperature is not supplied by the equipment used. Only the setpoint temperature is available.

In order to evaluate the influence of aging time, the non-used powder of a build was recovered, sieved, and reused for the next build. A total of 5 build cycles were studied, and each build cycle takes 4 h and corresponds to 4 h of cumulative build time.

Differential Scanning Calorimetry (Pyris Diamond from Perkin Elmer, Massachusetts, USA) thermograms were carried out in order to assess the influence of flame retardants and reuse cycles on the processing window. Heating and cooling cycles were performed between 50 and 210 °C at 10 °C/min. Melting and crystallization events were studied for the first and second heating and intermediate cooling stages. Tests were carried out on the powder blends and the square specimens produced by LS. To ensure the repeatability of characterization tests, the powder was collected at the end of the print job and sieved before being picked from three different areas after mixing. Three tests were made for each composition.

To evaluate sample porosity, both the density of powder blends and parts were determined using a helium pycnometer (AccuPyc 1330 from Micromeritics, Norcross, GA, USA). The internal porosity ϕ was calculated according to Equation (1):

(1) $$\phi \left(\%\right)=\frac{\rho s}{\rho p}\times 100$$

where ρp and ρs are the density of the powder blend and the printed part, respectively.

The microstructure of samples was studied using an environmental scanning electron microscope (Quanta 200 FEG from FEI Company, Hillsboro, OR, USA) equipped with an energy-dispersive X-ray spectroscope (Oxford INCA Energy System, Saclay, France) for the composition determination. The purpose of this observation was to assess the dispersion of flame retardants into PA 12 and the formation of voids for the different compositions and build cycles. In order to perform these observations, cryofracture using liquid nitrogen was carried out on specimen sheets produced by LS. Observations were made on the cross-sectional area of the cryofractured parts.

Thermal stability of powders and build specimens were measured using thermogravimetric analysis (SETSYS Evolution from SETARAM, Caluire, France) under nitrogen flow (40 mL/min) from 30 °C to 900 °C at 10 °C/min.

PCFC (Pyrolysis Combustion Flow Calorimeter) is a technique that allows the fire behavior of materials to be performed at the microscopic scale [27]. A PCFC from Fire Testing Technology Ltd. (East Grinstead, UK) was used to evaluate the fire behavior of samples at the microscale. Experiments were carried out on square sheets specimens according to method A of the ASTM D7309 using an FTT apparatus with a heating rate of 1 °C/min and a maximum pyrolysis temperature of 750 °C with a combustion temperature of 900 °C. The flow inside the combustor was a mixture of O_2_/N_2_ with a ratio of 20/80 at 100 cm^3^·min^−1^, and the sample weight was 2.5 ± 0.5 mg. The peak of rate release rate (pHRR) and corresponding time, total heat released (THR), were determined.

Fire behaviors were investigated according to ISO 5660-1 standard [28]. A cone calorimeter from Fire Testing Technology Ltd. (East Grinstead, UK) was used with an irradiance of 35 kW/m^2^ in order to carry out measurements on LS plates.

The following parameters were determined:

- Time to ignition (TTI)
- Peak of rate release rate (pHRR)
- Mass loss rate
- Total heat released (THR)
- Maximum of average rate of heat emission (MARHE) as a function of time.

PA 12 powder from all batches was analyzed using a Vertex 70 single reflection diamond ATR-FTIR (Bruker Corporation, Ettlingen, Germany). The wavelength range was in the 4000–400 cm^−1^ range. The baseline-subtracted spectra for each sample collected at various exposure times were recorded with 32 scans and a resolution of 2 cm^−1^.

## 3. Results and Discussions

### 3.1. DSC Thermograms

#### 3.1.1. Analysis of Initial and Reused Powder Blends

**Figure 2:**
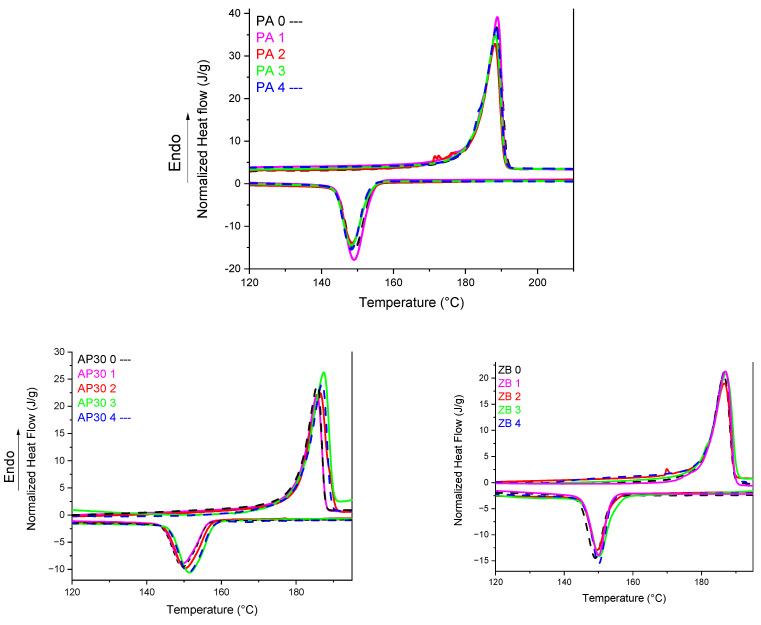
DSC thermograms of powder blends as a function of reuse.

Based on Figure 2 and Table 1, it appears that there are no significant changes while reusing PA 12 up to 4 times. The onset of first melting and crystallization temperatures always appear in the 182–183 °C and 153–154 °C ranges, respectively. PA 12 shows a sharp and regular endothermal peak for all cycles, which means the formation of very regular and homogeneous crystallites. It can be noted that the initial and reused powders exhibit high values of fusion enthalpies and, consequently, crystallization rates close to 40%. It can be noted that the crystallization enthalpy is much lower.

Our results are not completely in accordance with the previous work of Gomes et al., who performed twelve reuse cycles [29]. These authors have noted a decrease in the melting enthalpy, whereas the melting temperature remained unchanged. Our results indicate no significant changes in the melting enthalpy. Nevertheless, the PA 12 used was different as well as the LS printer.

The lack of differences after reuse can be explained by the fact that the aging time was not long enough to significantly modify the average molar weight of PA 12 inside the build chamber. Wudy et al. [12] have shown that it requires around 25 h of aging to see the first changes in the crystallization behavior of PA 12. The results are also linked to the type of LS printer used, as shown by the same authors. Duddleston et al. [30] also have reported that the differences in melting behavior of PA 12 as a function of aging are moderate for aging periods up to 24 h at 170 °C but significant for higher durations.

PA 12 powders with 30 wt% of ammonium polyphosphate (APP) show a slightly restrained sintering window of 25 °C compared with 30 °C for neat PA 12 samples. However, there are no significant differences between the thermal parameters. The melting temperature peak for the first heating is slightly shifted to higher temperatures. It can be ascribed to heat transfer phenomena between powders inside the DSC crucibles. It can be noted that the crystallization temperature is shifted to higher temperatures [31]. This seems to indicate that APP particles could act as nucleating agents.

PA 12 powders with 30 wt% of zinc borate show no onset temperature shifts in comparison with PA 12. The sintering window was slightly reduced compared with APP. Nevertheless, it can be seen that the peak melting temperature has also shifted to higher values. A slight increase in the peak temperature of crystallization occurs, indicating a limited nucleating role of zinc borate particles.

#### 3.1.2. Analysis of Specimens as Function of Reuse Cycles and Compositions

**Figure 3:**
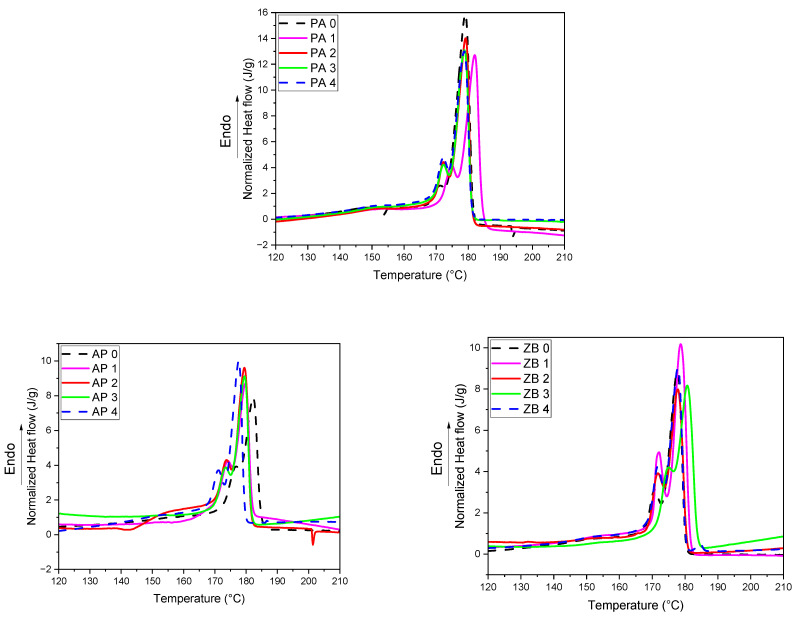
DSC thermograms of specimens as a function of reuse.

DSC experiments performed on specimens show that there are some differences between thermal parameters as a function of the reuse cycles (Figure 3, Table 2). The main difference between the behavior of the powders and the behavior of the powders is the presence of shoulders or secondary peaks of melting for all compositions. This can be ascribed to the formation and coexistence of different types of crystallites during the LS process. It has been reported by Schmid [32] that both α and γ structures could crystallize in PA 12 used in the LS process. PA 12 usually crystallizes in an γ structure. Its unit cell is monoclinic (pseudo-hexagonal) with four monomeric units in the unit cell, and the space group is P21/c. The structure exhibits a distortion of the amide group regarding the planar chain conformation, which is rather similar to γ-nylon 6 [33]. The α structure corresponds to a triclinic lattice with a planar and elongated zig-zag arrangement of polymer chains. According to Majewski et al. [34], it can also be considered that a fraction of the initial crystallites in the powders did not melt during the LS process as a function of the reuse cycle and process parameters. Due to the local heterogeneity of the material, there is no tendency to consider the location of the additional peaks or shoulders as a function of the reuse cycles. In addition, it can be noted that the crystallization enthalpies present some differences without significant tendency.

The main peak and offset crystallization temperatures are shifted to higher values in the presence of APP and ZB, also indicating a nucleation effect of both components. The crystallinity of zinc borate specimens is slightly lower (around 6%) than those of pure polyamide specimens. This can be explained by the different behavior of the materials submitted to the laser beam. Due to the presence of zinc borate particles, the penetration of the laser through the particle bed could be reduced. Consequently, the fraction of PA 12 that does not melt could be increased, and thereby, the crystallization/recrystallization phenomena differed between filled and unfilled specimens. Moreover, it can be noted that APP-filled specimens exhibit a higher crystallinity than unfilled samples. This can also be explained by a modification of heat transfer inside the powder bed due to the incorporation of FR particles.

In all cases (compositions and reuse cycles), the crystallization peak is regular and seems to correspond to a homogeneous crystallization process. Nevertheless, a second heating of the materials clearly shows the presence of two melting peaks (Figure S1, Table S1), which seems more homogeneous than the first melting.

### 3.2. Porosity of Specimens

A porous fraction was observed in PA 12 specimens, as expected, since a porosity value of around 5% is usual for LS processing of this material (Table S2). There were no significant changes in density while reusing PA 12 powder various times to process specimens. Moreover, the reduction in porosity with zinc borate can be ascribed to their smaller particle size in comparison with those of PA 12, which allows them to fill the voids between PA 12 particles, thereby increasing the compacity of the specimen. Moreover, the main mode of action of zinc borate as FR is the formation of a glassy structure when submitted to an intense heat source. Hence, it can be assumed that the strong intensity of the laser beam could cause a viscous flow of zinc borate, also contributing to reducing the porosity.

The density was also unchanged while using either ZB or APP after several reuse cycles. For ZB samples, its value was around 3%, whereas, for APP, the porosity was slightly higher than for neat PA 12, with a value of around 5.5%.

### 3.3. EDX of Powders

**Figure 4:**
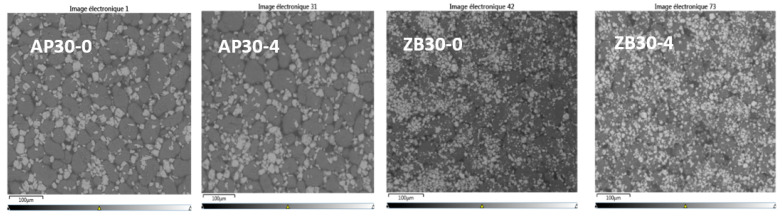
SEM images of filled powders. Virgin powder: AP30-0 and ZB30-0; 5 reuse cycles powder: AP30-4, ZB30-4.

SEM pictures of PA 12 powders are shown in Figure 4, and the results are presented in Table 3. The different FR particles can be easily identified. For both FRs, it appears that the particles are well dispersed with no apparent aggregates or clusters with virgin powder blend. After five cycles, AP remains well dispersed. Moreover, ZB particles seem to agglomerate after five reuse cycles, indicating some filler interactions.

### 3.4. Properties of LS Parts

#### 3.4.1. Scanning Electron Microscopy (SEM)

**Figure 5:**
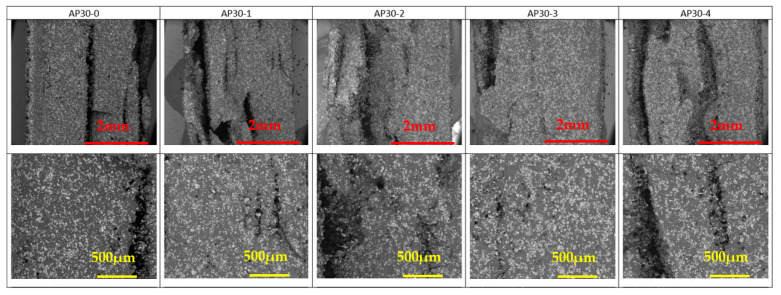
SEM observations of AP30 samples as a function of reuse. Mag ×60 (**top**) and Mag ×150 (**bottom**). White and brililiant areas represent AP particles, while dark areas represent PA 12.

**Figure 6:**
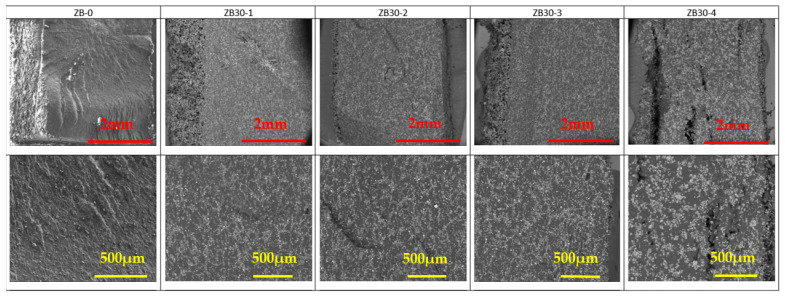
SEM observations of ZB30 samples as a function of reuse. Mag ×60 (**top**) and Mag ×150 (**bottom**). White and brilliant areas represent ZB particles, while dark areas represent PA 12.

**Figure 7:**
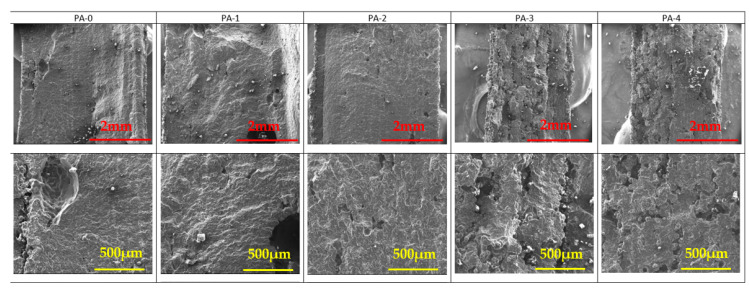
SEM observations of PA samples as a function of reuse. Mag ×30 (**top**) and Mag ×100 (**bottom**).

The SEM pictures of the cryofractured cross-section of PA 12 and PA 12 with FR particles are presented in Figure 5, Figure 6 and Figure 7. AP and ZB particles can easily be distinguished inside the PA 12 matrix, appearing as a brilliant white color.

A good distribution of the FR particles can be noted in Figure 5 and Figure 6 despite a tendency to aggregate with reuse stages. This will ensure that the fire reaction of the specimens will be homogeneous and effective in case of exposition to a heat source.

AP particles are well dispersed inside the PA 12 parts made by LS. While recycling AP30, there are no notable differences except a slight aggregation depending on the cumulative build time (Figure S2).

ZB particles are also well dispersed. Moreover, as a function of the reuse cycle, ZB particles seem to start to agglomerate with clusters, as observed in Figure 6 and Figure S3.

The morphology of flame retardants is nodular. So, there will be no mechanical reinforcement of PA 12 due to FR particles. Moreover, since their surface is not chemically modified, it can be supposed that the interfacial cohesion between the FR and the polymer matrix is low, leading to a reduction in the tensile strength of the composites.

It appears clear in Figure 7 that pure PA 12 specimens exhibit a higher porosity than specimens with FR. Moreover, it seems that the fusion between particles becomes more difficult with the reuse stages due to the increasing heterogeneity of the material.

#### 3.4.2. Thermal Stability (Thermogravimetric Analysis—TGA)

**Figure 8:**
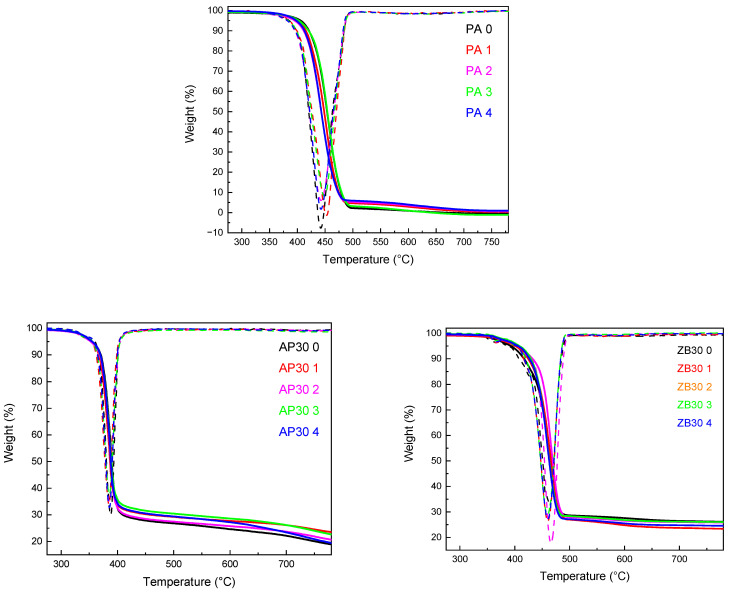
TGA thermograms of powders as a function of reuse.

The influence of reuse on the thermal stability of PA 12 and PA 12 with AP30 or ZB30 powder blends was evaluated by TGA under a nitrogen atmosphere. The results are shown in Table 4. Three tests were carried out for each sample to ensure repeatability. The mean value of the three tests was then recorded. As can be observed in Figure 8, neat PA 12 degrades in one steep between 380 and 490 °C with no residue at higher temperatures, which is in agreement with the well-known thermal degradation of polyamides [25]. Reuse has a slight influence on characteristic degradation temperatures, with an increase in T5% and a decrease in T_20%_ and T_50%_. These differences can be attributed to the laser sintering process, which may promote the decomposition of the PA 12 chain backbone, lowering characteristic decomposition temperatures [10]. It has been seen in some studies dealing with the thermal degradation of PA 12 that the gas released is mainly composed of CO, CO_2_, hydrocarbons, and dodecaprolactam [35].

The addition of ammonium polyphosphate leads to a strong decrease in T_5%_, T_20%_, and T_50%_ due to the earlier degradation of AP with the release of water and ammonia at lower temperatures [23], giving condensed phosphoric acid, which may induce the scission of alkylamide group [35].

The amount of residues is found to be between 20 and 23%. Using APP will lead to faster thermal degradation than with neat PA 12. The mass of residues is mainly due to the initial amount of APP. It has been seen that in the same temperature range as PA 12 (300–750 °C), pure APP is losing around 31% of its initial mass [23], then we can calculate the theoretical residue as follows:

$$30\left(w\%\;of\;APP\right)\times 0.69\;\left(mass\;obtained\;of\;pure\;APP\;at\;750\;{}^{\circ}C\right)=20.7\%\;(theoretical\;residue)$$

Reuse of compositions containing AP leads to a similar evolution of behavior as neat PA 12, with a small increase in T_5%_ and a decrease in T_20%_ and T_50%_, indicating a negligible influence of the flame retardant on the thermal stability of PA 12 during the LS process.

Unlike AP30, ZB confers better thermal stability to PA 12 with an onset of degradation and a maximum degradation temperature that is slightly higher than neat PA 12. T_5%_, T_20%_ and T_50%_ are also higher. Zinc borate starts to decompose at about 370 °C with release of water, leaving about 85% of inorganic residue [26].

The mass loss is related to the loss of water from the initial composition of the FR: 2ZnO•3B_2_O_3_•3.5H_2_O [26] that occurs starting from 370 °C and ending at 410 °C. The amount of residue is calculated as below for 30ZB:

$$30\left(w\%\;of\;ZB\right)\times 0.88\;\left(mass\;obtained\;of\;pure\;ZB\;at\;750\;{}^{\circ}C\right)=26.4\%\;(theoretical\;residue)$$

The results in Table 4 show that the experimental values are slightly below the estimated values. Consequently, the obtained values are not dependent on the number of cycles of reuse.

#### 3.4.3. Fire Behavior at Microscopic Scale—PCFC

**Figure 9:**
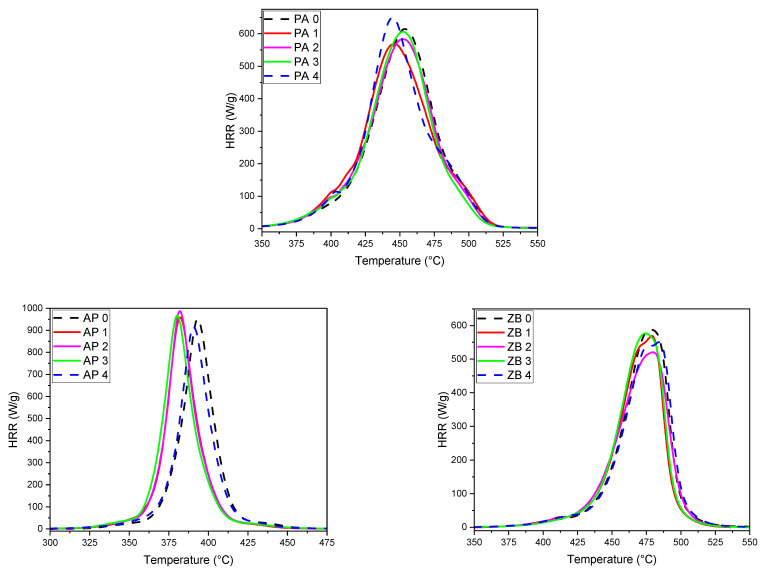
PCFC thermograms of PA 12 samples (**top**), with 30 wt% AP (**bottom left**) and 30 wt% ZB (**bottom right**).

Figure 9 presents the heat release curves as a function of temperature for pure PA 12 and compositions with flame retardants. All corresponding data are presented in Table 5. The HRR curve for pure PA 12 exhibits two shoulders on both sides of the peak. This can be explained by the presence of a wide range of chain lengths as well as the possible formation of crosslinked structures resulting from the LS process.

The results related to the first cycle also show that the HRR peak for pure PA 12 is shifted to lower temperatures for APP composition and higher temperatures for ZB composition. As usually observed, the HRR curves correspond with the differential curves of TGA (Figure 8).

In addition, the peak corresponding to APP composition is lower than this of pure PA 12, whereas the reverse is noted for the composition with ZB.

This can be explained by the specific flame-retardant activity of each FR. APP decomposes at a lower temperature than many polymers in order to promote charring by phosphorylation reactions. These reactions release water and ammonia, which leads to hydrolysis and aminolysis reactions and a strong release of short polymer chains, which will feed the gaseous phase in volatile combustibles [36]. This accounts for the highest value of pHRR. Conversely, the action mode of zinc borate is based on the endothermal release of water and the formation of a glassy barrier structure [37]. This leads to a slower release of volatile combustibles in the gas phase and a lower pHRR. However, despite the use of flame retardants, it can be noted that THR values do not change significantly when the amount of FR in PA 12 is considered.

The study of the specimens processed with the reuse powders always shows the same structure as the initial ones for PA 12. The evolution towards a more heterogeneous structure of the PA 12 can be seen from a decrease in the height of the peak for a rather constant value of THR. In the case of APP and ZB compositions, the shoulders are less present, and few evolutions have been noted. The presence of FR tends to level off the role of the heterogeneity of the polymer. However, two joint peaks appear for the last reuse cycles with ZB, showing that the heterogeneous behavior of PA 12 seems enhanced by the presence of the FR.

#### 3.4.4. Fire Behavior—Cone Calorimeter

**Figure 10:**
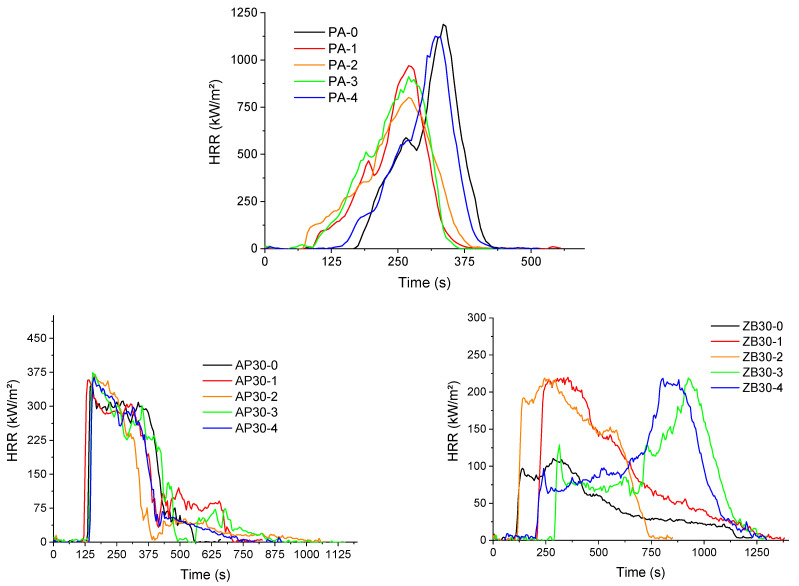
Cone calorimeter curves of formulations as a function of reuse.

Cone calorimeter results of PA 12, AP30, and ZB30 are shown in Figure 10, and complete data are given in Table 6. PA 12 seems to exhibit a typical behavior of neat polymers, but a double peak of Heat Release Rate is observed with a maximum value of 1416 kW/m^2^.

Some important differences are observed when powders are reused. Despite the fact that the evolution is not monotonous, a decrease in TTI always occurs, which confirms the formation of short chains resulting from chain scissions and, consequently, aging processes.

Moreover, a decrease in pHRR is observed after the first reuse, which can indicate a more heterogeneous structure for PA 12 with possibly crosslinked chains. The addition of flame retardants leads to a decrease of about 75% and 92% of pHRR for AP30 and ZB30 formulations, respectively.

According to the action mode mentioned previously, the addition of ammonium polyphosphate leads to a decrease in TTI due to the release of short polymer chains resulting from chain scissions. Due to the formation of a charred structure, pHRR is significantly reduced, and a residue of 30% of the initial mass is formed. The HRR curves corresponding to the reused material and the initial one are very similar. Consequently, the expected evolution of the PA 12 due to aging seems here to be leveled due to the presence of the FR.

The addition of zinc borate also leads to a stronger decrease in pHRR (pHRR value around 125 kW/m^2^) when virgin powders are used. TTI is similar to the value obtained for pure PA 12. A high value of residue is noted, around 40%.

Interestingly, the profile of HRR curves shows important differences as a function of reuse cycles. For the first and second reuses, HRR increases strongly in comparison with the values observed for the initial composition, with a pHRR value higher than 200 kW/m² at a time of around 300 s. This can be ascribed to a predominance of chain scissions over other aging mechanisms. Conversely, for the third and fourth reuses, a first peak appears at around 250–300 s, followed by an HRR decrease and a plateau, which can be ascribed to the formation of a surface protective layer, according to Hull & Schartel [38]. Then, this layer breaks at longer times, and the pHRR is postponed to higher times (around 900–1000 s). Hence, PA 12 seems more stable, and ZB is more effective for these last compositions than the first ones. This can be explained by a possible predominant crosslinked structure for PA 12 but also by the formation of a network of agglomerated fillers (with possibly the formation of glassy structures), as shown in Figure 4, which could ensure a better mechanical cohesion of the protective layer formed during the thermal degradation of the material.

#### 3.4.5. Fourier Transform Infrared Spectra of LS Samples

**Figure 11:**
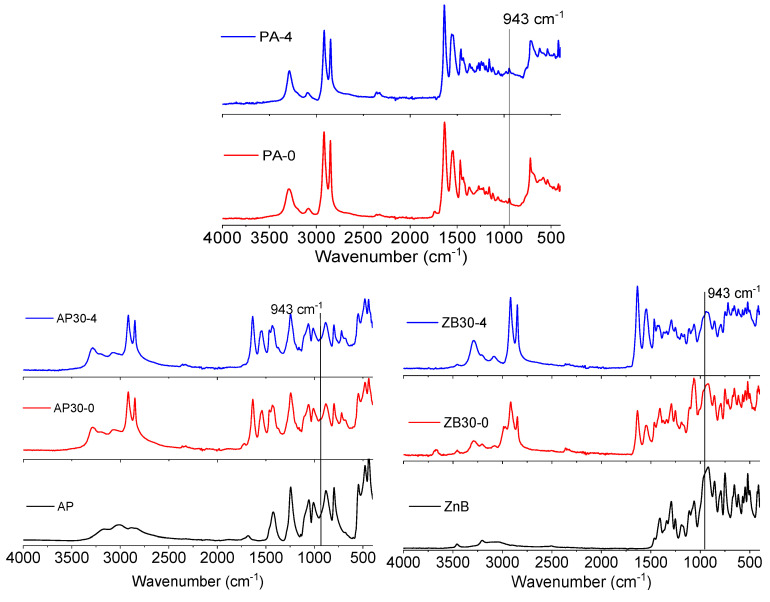
FTIR spectra of the virgin and four cycle PA 12, AP30, and ZB30 powders.

In order to assess the influence of flame retardants on the aging of polyamide 12, FTIR/ATR analyses were conducted on printed samples. The results are shown in Figure 11. Virgin PA 12 presents the characteristic vibration bands that can be assigned to NH stretching (3293 cm^−1^), Fermi resonance of NH stretching (3091 cm^−1^), asymmetric and symmetric CH2 stretching (2918 and 2848 cm^−1^), carbonyl group (1740 cm^−1^), amide I stretching (1633 cm^−1^), amide II stretching (1545 cm^−1^) and CO-NH in plane (946 cm^−1^). These bands usually correspond to the α crystalline phase of PA 12. However, after four reuses, some differences can be observed, in particular, the disappearance of the 1740 cm^−1^ band, which can be assigned to post-condensation reactions.

**Figure 12:**
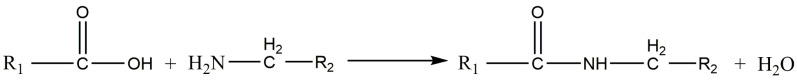
Post-condensation reaction (Polyamide 12).

The reaction below (Figure 12) shows the equilibrium between the concentrations of amide groups and water at the chain ends of polyamides. The dry conditions promoted by the LS process shift the equilibrium of the chemical reaction towards the products by continuous water removal and lead to an increase in molecular weight.

AP30 formulation presents, in addition to PA 12 bands, characteristic bands of ammonium polyphosphate, corresponding to N–H (centered at 3020 cm^−1^), P=O (1244 cm^−1^), symmetric stretching of P–O (1060 cm^−1^), symmetric vibration of PO_2_ and PO_3_ (1010 cm^−1^), asymmetric stretching of P–O (880 cm^−1^), and P–O–P (798 cm^−1^) [39,40]. Interestingly, the only difference after four reuse cycles is the disappearance of the 1740 cm^−1^ band.

Similarly, ZB30 formulations present the characteristic vibration bands of zinc borate that can be assigned to the presence of stretching vibrations of OH groups (3460 cm^−1^). The bands between 1410 cm^−1^ and 1253 cm^−1^ can be assigned to the asymmetric stretching of trihedral borate groups, and the band at 1062 cm^−1^ can be attributed to the asymmetric stretching of BO_4_ [41]. Moreover, the band at 1740 cm^−1^ disappears from the first reuse, indicating faster post-condensation reactions.

#### 3.4.6. Polyamide 12 Phase Analysis—XRD

The diffractograms of pure zinc borate and zinc borate irradiated by the LS CO_2_ laser beam are shown in Figure S4. There are no differences between pure zinc borate and zinc borate irradiated by the CO_2_ laser. One can highlight from the diffractograms that the irradiated crystals are slightly higher than non-irradiated ones. Zinc borate far from the irradiation area (ZB SLS) also shows no difference from zinc borate, which has been directly impacted by the laser beam. This proves that there was no lattice distortion when zinc borate was used as a flame retardant with PA 12.

## 4. Conclusions

Flame retardant compositions of PA 12 with ammonium polyphosphate and zinc borate were processed using the LS additive manufacturing technology. The fire performance of these compositions was evaluated using PCFC and cone calorimeter. Moreover, the influence of the aging of powders inside the LS printer on the characteristics of the pure PA 12 powders and powder blends with flame retardants, as well as on the fire behavior of resulting materials, was studied. Investigations about aging were performed through four cycles of powder reuse.

DSC experiments showed a high level of crystallinity for the PA 12 powders, which was maintained after all the reuse cycles. On the whole, it can be considered that the aging time was not long enough to cause variation in the thermal characteristics of pure PA 12 powders determined using this technique. The presence of flame retardants tends to restrict the processing window. Crystallization temperatures were shifted to higher values, showing a nucleating effect on APP and ZB. DSC performed on samples processed through LS showed through the melting behavior the presence of different types of crystallites for the initial and all the reuse cycles (possibly coexistence of α and γ crystalline structures) and globally a loss of crystallinity rate in comparison with the powders.

The heterogeneous behavior of the different types of materials processed using LS is also witnessed by the PCFC curves, which showed the decomposition of different structures, possibly chains of diverse lengths because of scission and post-condensation phenomena, crosslinked polyamide. The action mode of the two types of flame retardants is well identified from PCFC and TGA curves, considering the modifications of thermal stability in comparison with pure PA 12.

Cone calorimeter tests highlight the influence of aging and the reuse of powders on fire behavior. The subsequent formation of short chains of PA 12 with reuse led to a decrease in time to ignition and to a reduction in the time of peak of heat release rate for pure PA 12. Regarding APP compositions, the effectiveness of the flame retardant led to a strong decrease in pHRR values and a leveling of the behavior for the different reuse cycles. The case of ZB is particularly interesting since the use of reused powders appears detrimental to the fire performance. In addition, it has been noticed that the HRR profile was very different between the first and last reuse cycles. Finally, it has been shown that aging phenomena occur when powders are reused. Changes in structures for these powders influenced the fire behavior, which has been investigated in this research work by the use of PCFC at the microscale and cone calorimeter at the mesoscale.

## Figures and Tables

**Table 1:** DSC data of neat polyamide 12 and blends of FR + PA 12 (AP30 for APP and ZB30 for ZB) powders. T_ic_ and T_im_ are onset temperatures of crystallization and melting, respectively. ΔH_c_ and ΔH_F_ represent the crystallization enthalpy and the melting enthalpy, respectively.

Sample	T_ic_ (°C)	T_c_ Peak (°C)	T_im_ (°C)	T_m_ Peak (°C)	ΔH_c_ (J/g)	Processing Window ΔT (°C)	ΔH_F_ (J/g)
PA-0	153.8	148.2	182.4	183.9	45.0	29.1	88.1
PA-1	154.1	148.8	182.3	183.4	46.0	28.7	83.3
PA-2	153.2	148.6	183.5	183.5	46.1	30.3	86.6
PA-3	153.2	148.4	182.5	182.5	45.4	29.3	85.6
PA-4	153.2	148.3	182.1	182.1	45.7	28.9	88.8
AP30-0	155.7	152.7	181.0	184.8	45.9	25.3	80.2
AP30-1	155.6	149.4	181.2	184.2	45.7	25.6	79.9
AP30-2	158.2	151.6	183.4	185.0	45.1	25.2	78.0
AP30-3	158.0	152.0	182.6	186.2	47.7	24.6	78.9
AP30-4	156.8	150.4	181.8	185.6	46.6	25.0	76.3
ZB30-0	153.0	148.5	180.5	186.0	47.8	27.5	76.8
ZB30-1	154.2	149.0	182.0	187.6	47.1	27.8	76.8
ZB30-2	154.5	150.0	182.6	188.2	47.7	28.0	76.5
ZB30-3	153.2	149.4	182.6	186.5	47.8	29.4	80.0
ZB30-4	153.0	150.5	181.9	187.1	46.8	28.9	78.2

**Table 2:** DSC data of specimens processed through LS of neat polyamide 12 and blends of FR + PA 12 for second heating. (AP30 for APP and ZB30 for ZB). T_ic_ and T_im_ are the onset of crystallization and the first melting stage, respectively.

Sample	T_ic_ (°C)	T_c_ Peak (°C)	T_im_ (°C)	T_m_ Peak (°C)	ΔH_c_ (J/g)	ΔH_F_ (J/g)
PA-0	152.6	148.7	173.3	183.3	50.2	58.0
PA-1	153.0	149.4	172.4	182.6	49.5	56.9
PA-2	153.1	149.3	173.0	181.9	49.7	57.0
PA-3	153.1	149.6	174.9	182.2	46.6	55.5
PA-4	154.0	149.8	176.7	184.2	49.2	50.9
AP30-0	159.2	153.4	175.9	182.9	45.5	57.3
AP30-1	158.9	153.8	177.1	183.3	40.1	54.9
AP30-2	158.6	153.7	176.4	184.4	42.5	57.7
AP30-3	158.5	153.6	177.3	183.1	41.1	58.4
AP30-4	158.8	154.0	177.1	183.1	42.4	56.5
ZB30-0	159.3	154.2	176.1	183.4	46.0	58.0
ZB30-1	160.1	152.7	175.1	183.6	46.8	46.3
ZB30-2	160.0	152.5	176.2	183.5	45.4	52.2
ZB30-3	160.0	153.2	175.8	183.0	47.7	49.8
ZB30-4	160.0	153.2	176.0	183.7	46.9	48.0

**Table 3:** EDX data of powders.

Sample	Theoretical P Content (%)	P (%)	Theoretical Zn Content (%)	Zn (%)
AP30-0	9.57	9.3 ± 0.3	-	-
AP30-1	9.57	9.6 ± 1	-	-
AP30-2	9.57	10.1 ± 0.8	-	-
AP30-3	9.57	10.4 ± 0.3	-	-
AP30-4	9.57	9.5 ± 0.4	-	-
ZB30-0	-	-	15.5	15.5 ± 0.5
ZB30-1	-	-	15.5	11.8 ± 2
ZB30-2	-	-	15.5	12.5 ± 1.3
ZB30-3	-	-	15.5	12.8 ± 1.2
ZB30-4	-	-	15.5	12.6 ± 0.9

**Table 4:** TGA data for polyamide 12 and FR.

Sample	T_5%_	T_20%_	T_50%_	Residue at 750 °C (%)
PA-0	395.2	432.8	451.3	0
PA-1	399.9	427.9	448.2	0.4
PA-2	396.0	424.9	443.7	0.7
PA-3	397.3	425.1	443.9	0.9
PA-4	398.7	425.4	443.7	1.0
AP30-0	354.1	378.5	389.9	20.0
AP30-1	352.3	374.7	386.5	22.5
AP30-2	352.6	376.6	387.4	21.9
AP30-3	355.2	377.5	389.0	23.0
AP30-4	355.0	377.0	388.0	20.9
ZB30-0	405.8	447.3	466.1	25.1
ZB30-1	398.9	441.3	463.6	23.5
ZB30-2	406.4	452.1	468.9	25.9
ZB30-3	408.1	443.7	462.5	26.1
ZB30-4	406.2	443.5	462.9	24.6

**Table 5:** PCFC data of neat PA 12, 30% ZB, and 30%AP.

Sample	THR (kJ/g)	pHRR (W/g)	Peak Temperature (°C)
PA-0	34.7	699	454.5
PA-1	33.6	573	460.4
PA-2	35.2	586	454.7
PA-3	34.2	575	466.6
PA-4	34.3	592	461.7
AP30-0	24.7	951	393.4
AP30-1	24.1	958	381.9
AP30-2	24.4	987	381.8
AP30-3	25.7	968	380.4
AP30-4	24.1	935	390.5
ZB30-0	24.5	588	477.3
ZB30-1	22.8	569	478.7
ZB30-2	23.8	521	479.8
ZB30-3	23.7	577	474.6
ZB30-4	24.0	550	483.8

**Table 6:** Cone calorimeter data of various formulations as a function of reuse cycle.

Sample	TTI (s)	pHRR (kW/m²)	THR (MJ/m²)	MAHRE (kW/m²)	Residue (%)
PA-0	154 ± 10	1416 ± 200	131 ± 5	337 ± 25	-
PA-1	94 ± 3	942 ± 37	117 ± 3	328 ± 7	-
PA-2	80 ± 5	893 ± 98	120 ± 1	345 ± 15	-
PA-3	88 ± 5	827 ± 95	115 ± 5	324 ± 23	-
PA-4	193 ± 20	1037 ± 102	115 ± 4	320 ± 15	-
AP30-0	122 ± 10	351 ± 5	92 ± 6	196 ± 4	31 ± 2
AP30-1	118 ± 3	347 ± 14	105 ± 5	197 ± 5	30 ± 3
AP30-2	144 ± 1	373 ± 2	81 ± 6	180 ± 10	27 ± 1
AP30-3	139 ± 3	363 ± 15	88 ± 8	192 ± 8	26 ± 1
AP30-4	143 ± 2	374 ± 14	86 ± 5	180 ± 10	27 ± 1
ZB30-0	115 ± 20	125 ± 15	95 ± 8	120 ± 10	38 ± 3
ZB30-1	214 ± 6	163 ± 70	92 ± 13	115 ± 15	35 ± 3
ZB30-2	189 ± 5	175 ± 61	98 ± 6	130 ± 14	33 ± 1
ZB30-3	252 ± 20	204 ± 19	101 ± 8	65 ± 8	25 ± 1
ZB30-4	180 ± 20	220 ± 22	95 ± 5	72 ± 5	29 ± 2

## Data Availability

All data are reported in the article.

## Supplementary Materials

The following supporting information can be downloaded at: https://www.mdpi.com/article/10.3390/ma17164064/s1, Figure S1: DSC data of second heating (LS specimens); Table S1: DSC data of second heating (LS specimens); Table S2: Porosity results in PA12, AP30 and ZB30 over cycle of reuse; Figure S2: AP30 particle’s aggregation with formation of a network. AP30-0 is represented in left side and AP30-4 in right; Figure S3: SEM images of ZB 0 (left) and ZB 4 (right); Figure S4: Diffractograms of (1) Pure zinc borate (2) Zinc borate that was inside build chamber without any irradiation by CO2 laser (3) Zinc borate that was inside build chamber and underwent CO2 laser irradiation.
