# Supplementary material for: Towards the Reuse of Fire Retarded Polyamide 12 for Laser Sintering
Source: Materials (Basel). 2024 Aug 15;17(16):4064. doi: 10.3390/ma17164064 (PMC11356142; doi:10.3390/ma17164064)
Supplement: Supplementary file 1 [file materials-17-04064-s001.zip › materials-3121529-supplementary.pdf]

Supplementary

Towards the Reuse of Fire Retarded Polyamide 12 for Laser Sintering

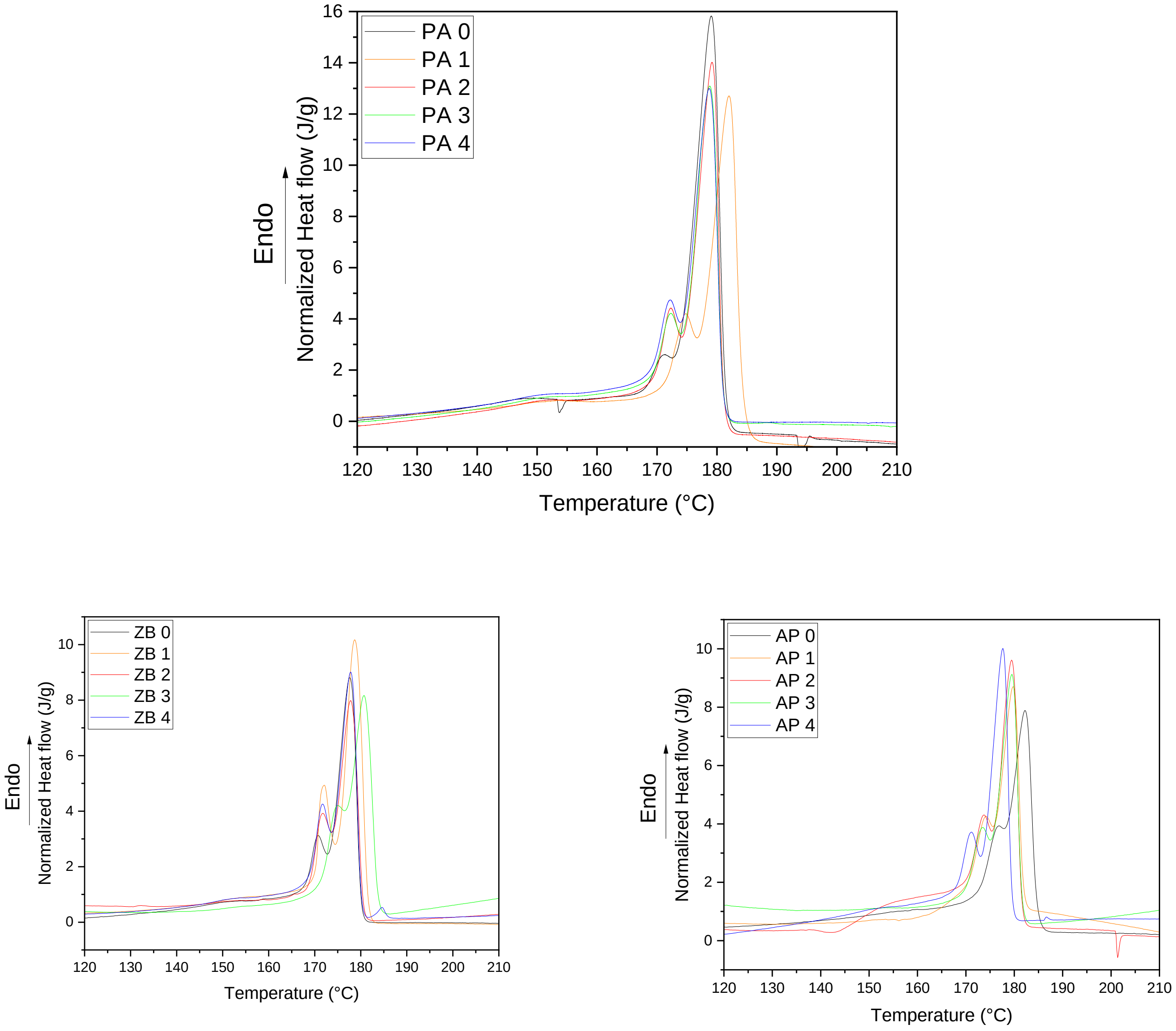

Figure S1. DSC of specimens (second heating) for PA 12 (top), AP (bottom left) and ZB (bottom right).

Table S1. DSC data of second heating (LS specimens).

Sample	T_{im}(°C)	T_{peak} (°C)	T_{endm} (°C)	Melting Enthalpy (J/g)	Crystallinity (%)
**PA 0**	174,7	179,8	182,1	37,59	17,97
**PA 1**	175,1	180,1	182,8	35,61	17,02
**PA 2**	174,8	179,9	182,5	35,14	16,80
**PA 3**	175,1	180,1	182,9	36,29	17,35
**PA 4**	176,2	181,7	183,5	35,47	16,96
**ZB 0**	175,5	180,4	182,6	35,67	17,05
**ZB 1**	175,7	181,4	183,4	34,71	16,59

**ZB 2**	175,3	180,9	183,8	34,87	16,67
**ZB 3**	174,8	180,9	183,3	33,62	16,07
**ZB 4**	175,4	181,2	183,9	34,14	16,32
**AP 0**	175,9	180,8	183,3	36,74	17,56
**AP 1**	176,4	181,4	183,9	37,91	18,12
**AP 2**	176,6	181,2	183,9	35,49	16,96
**AP 3**	176,5	181,2	183,6	37,84	18,09
**AP 4**	176,4	181,2	183,5	37,39	17,87

Helium pycnometer : porosity

Table S2. Porosity results in PA12, AP30 and ZB30 over cycle of reuse.

Sample	Part density (g/cm³)	Powder density (g/cm³)	Porosity (%)
**AP30 0**	1,18	1,23	4,1
**AP30 1**	1,19	1,23	4,0
**AP30 2**	1,19	1,24	4,0
**AP30 3**	1,2	1,24	3,2
**AP30 4**	1,2	1,24	3,2
**ZB30 0**	1,27	1,31	3,1
**ZB30 1**	1,27	1,3	2,3
**ZB30 2**	1,27	1,29	1,6
**ZB30 3**	1,27	1,3	2,3
**ZB30 4**	1,27	1,3	2,3
**PA 0**	1,01	1,07	5,6
**PA 1**	1,01	1,06	4,7
**PA 2**	1,01	1,06	4,7
**PA 3**	1,01	1,06	4,7
**PA 4**	1	1,06	5,7

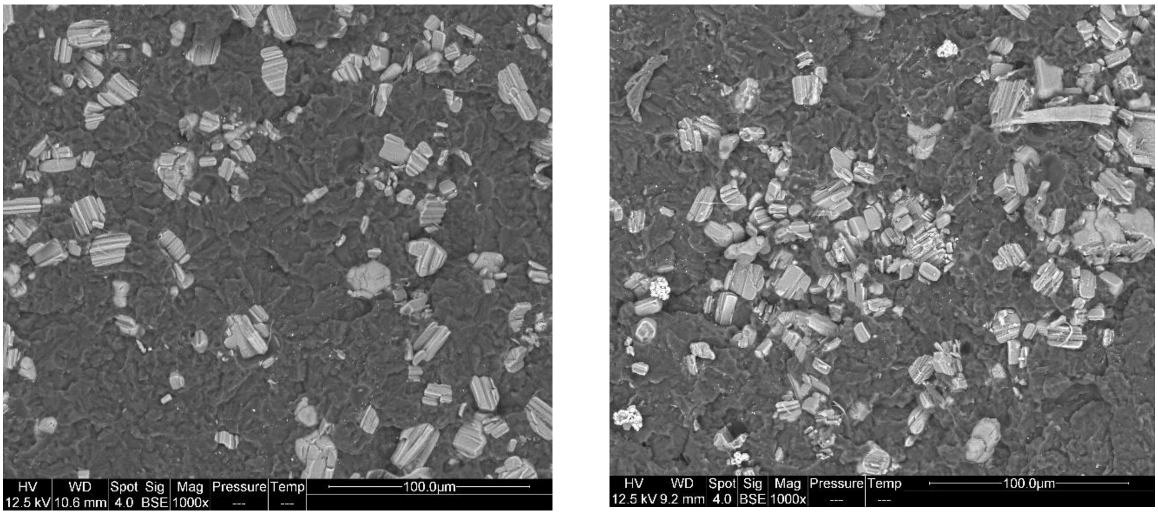

Figure S2. AP30 particle's aggregation with formation of a network. AP30-0 is represented in left side and AP30-4 in right.

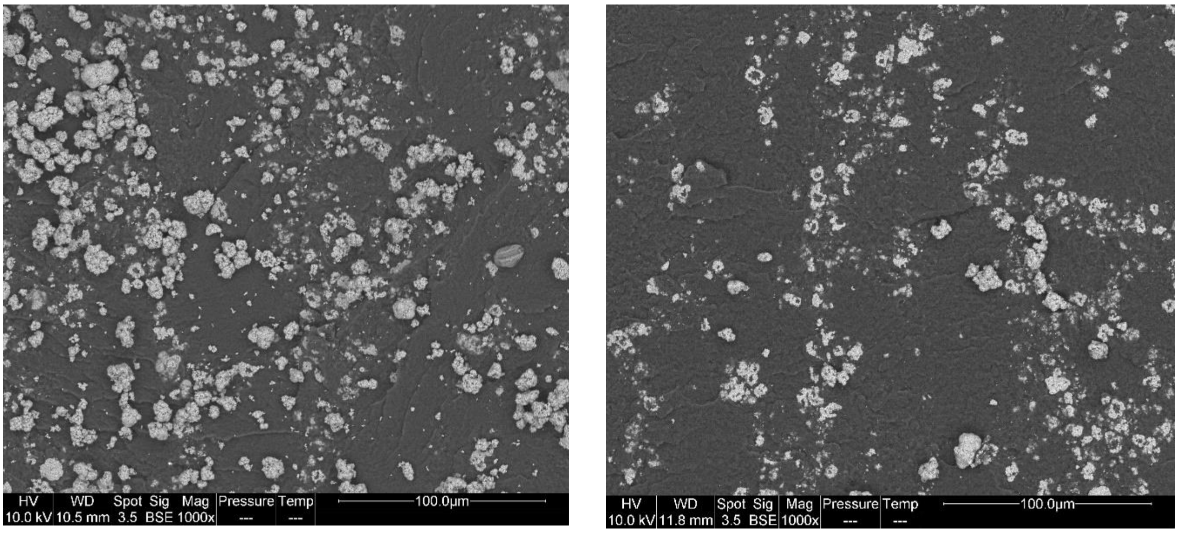

Figure S3. SEM images of ZB 0 (left) and ZB 4 (right).

XRD of powders : Influence of CO2 laser and cycle of reuse.

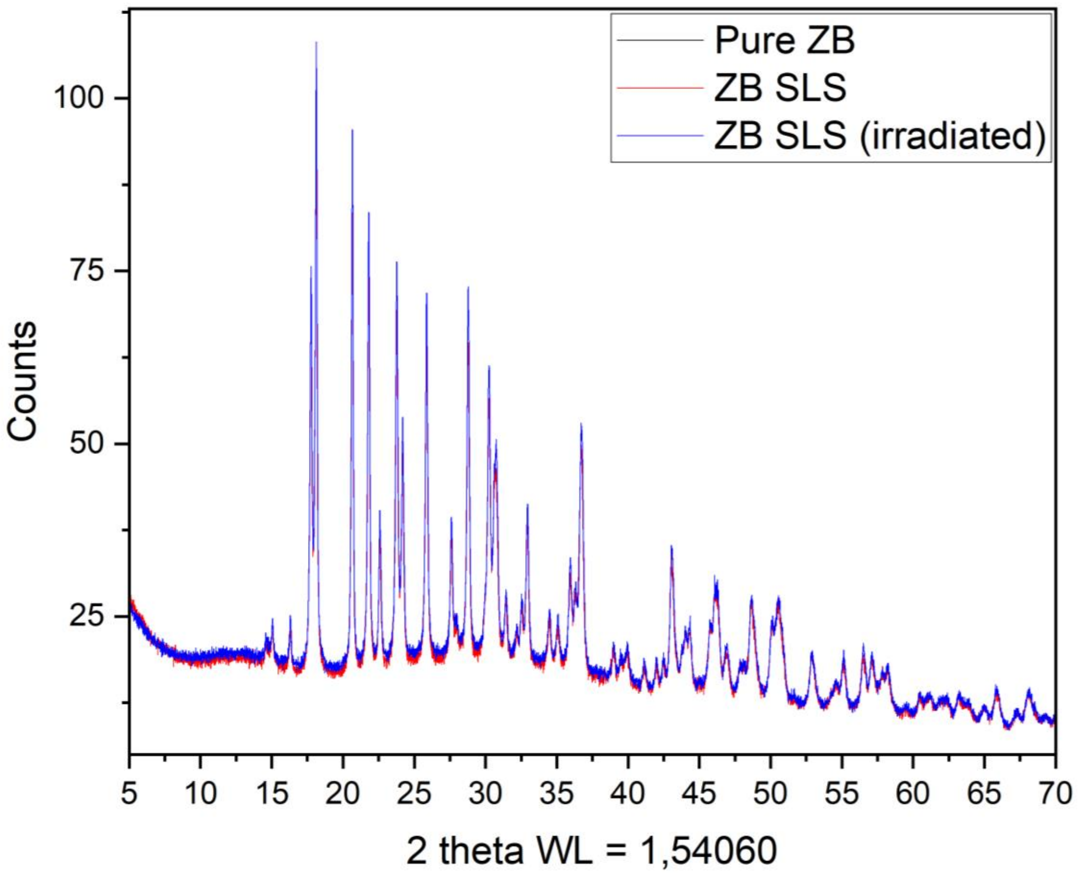

Figure S4. Diffractograms of 1) Pure zinc borate 2) Zinc borate that was inside build chamber without any irradiation by CO_2 laser 3) Zinc borate that was inside build chamber and underwent CO_2 laser irradiation.
